# Weekly *versus* three weeks chemotherapy for advanced ovarian cancer: a meta-analysis

**DOI:** 10.18632/oncotarget.11094

**Published:** 2016-08-05

**Authors:** Claudia Marchetti, Francesca De Felice, Angela Musella, Innocenza Palaia, Marco Monti, Daniela Musio, Ludovico Muzii, Vincenzo Tombolini, Pierluigi Benedetti Panici

**Affiliations:** ^1^ Department of Gynecological and Obstetrical Sciences and Urological Sciences, “Sapienza” University of Rome, Rome, Italy; ^2^ Department of Radiotherapy, Policlinico Umberto I “Sapienza” University of Rome, Rome, Italy

**Keywords:** ovarian cancer, dose-dense, carboplatin, paclitaxel, weekly

## Abstract

**Aim:**

Three weeks paclitaxel and carboplatin has been considered the standard of care for primary treatment of ovarian cancer (OC). Whether weekly therapy will further improve the clinical outcomes or not is still unclear. We conducted a meta-analysis to compare the two regimens.

**Method:**

Articles were selected with a systematic approach, using PubMed databases. Trials concerning comparison between carboplatin plus weekly paclitaxel (dose-dense regimen) and carboplatin plus paclitaxel every 3 weeks were considered. Outcomes included overall survival (OS), progression free survival (PFS) and severe acute toxicity.

**Results:**

Dose-dense regimen was associated with significant improvement of PFS compared with standard schedule, with HR of 0.73 (95% CI 0.61-0.88, *p* = 0.001). There was no difference in OS between treatment regimens (HR 0.95, 95% CI 0.77-1.16, *p*=0.06), as well as in term of severe acute toxicity.

**Conclusion:**

Dose-dense regimen is superior to standard schedule in terms of PFS. Further studies are necessary to firmly confirm this evidence in advanced OC treatment.

## INTRODUCTION

Paclitaxel and carboplatin regimen is considered the standard of care for patients with newly diagnosed ovarian cancer (OC) [1]. It is recommended an intravenous infusion of 3-hour paclitaxel 175 mg/m^2^ and carboplatin at an area under the curve (AUC) of 6 mg/ml/min repeated every 3 weeks for six cycles. In attempt to improve clinical outcomes, based on the efficacy demonstrated in breast cancer [2], new schedules have been tested, including dose-dense regimen. Generally it consisted of weekly paclitaxel plus carboplatin given every 3 weeks. Doses depend on trials and range from paclitaxel 60 mg/m^2^ to 90 mg/m^2^ and carboplatin AUC 2 mg/ml/min to AUC 6 mg/ml/min [3–6]. However, at present, there is no data indicating which regimen is superior.

The main purpose of this meta-analysis was to compare the efficacy of weekly *versus* 3 weeks chemotherapy regimes, in term of survival outcomes and toxic effects.

## RESULTS

### Search results

**Figure 1:**
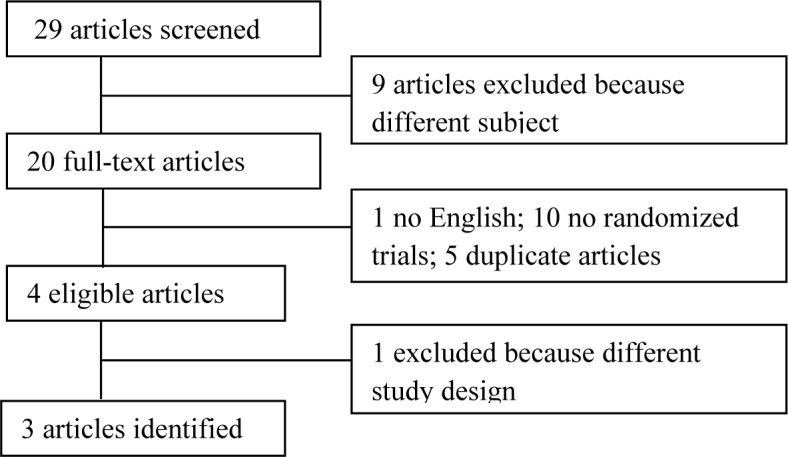
PRISMA flow diagram

The meta-analysis included randomized phase III trials, only. Figure 1 shows the study selection process.

The literature search identified a total of 29 potentially relevant articles. Articles were excluded because the subject matter was not related to the study (*n* = 9), or the trial was not randomized phase III (*n* = 3), or it was a review (*n* = 7), or the article was not published in English (*n* = 1) or they were duplicate articles (*n* = 5).

To avoid bias and reduce heterogeneity, van der Burg trial was excluded, due to double randomization performed in the study design [6]. At the end of the review process, 3 trials were eligible (2133 patients) comparing carboplatin plus weekly paclitaxel *versus* carboplatin plus paclitaxel every 3 weeks [3–5].

In one trial (Gynecologic Oncology Group (GOG) 0262, [5]) patients in either group could opt to receive bevacizumab. For survival analysis, update published follow-up data were considered for one trial [11]. Main characteristics of the trials are shown in Table 1.

**Table 1:** Trials' characteristics

	Trial			Patients		Stage disease			Schedule		
Author	Name	Phase	Year	Total	Per arm	I-II	III	IV	Regimen	Drug and dose	Follow-up (median)
Katsumata	JGOG 3016	III	2013	631	312	62	202	48	weekly	Paclitaxel 80 mg/mq; carboplatin AUC 6 mg/mL per min*	76.8 months
					319	54	215	50	3-weeks	Paclitaxel 175 mg/mq; carboplatin AUC 6 mg/mL per min	
Pignata	MITO 7	III	2014	810	406	62	234	110	weekly	Paclitaxel 60 mg/mq; carboplatin AUC 2 mg/mL per min	22.3 months
					404	58	255	91	3-weeks	Paclitaxel 175 mg/mq; carboplatin AUC 6 mg/mL per min	
Chan	GOG 0262	III	2016	692	346	8	241	97	weekly	Paclitaxel 80 mg/mq; carboplatin AUC 6 mg/mL per min*	28 months
					346	10	223	113	3-weeks	Paclitaxel 175 mg/mq; carboplatin AUC 6 mg/mL per min	
				Subgroup							
					55				weekly	without bevacizumab	
					57				3-weeks	without bevacizumab	
					219				weekly	plus bevacizumab 15 mg/Kg*	
					225				3-weeks	plus bevacizumab 15 mg/Kg*	

* administered intravenously every 21 days

AUC: area under the curve

### Progression free survival

**Figure 2:**
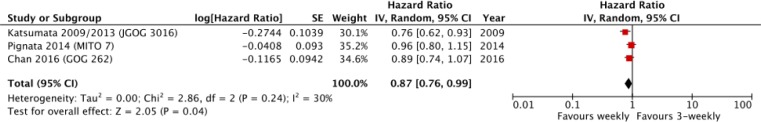
Progression free survival of overall cohort

Overall, PFS after weekly schedule was significantly better than PFS after 3-weekly regimen (HR 0.87, 95% CI 0.76-0.99, *p* = 0.04; I^2^ 30%; Figure 2).

**Figure 3:**
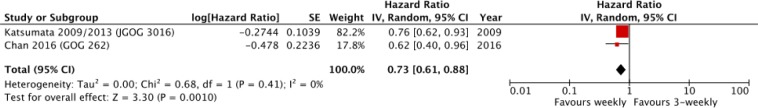
Progression free survival: Subgroup analysis in trials comparing weekly paclitaxel plus carboplatin (AUC 6 mg/mL per min) with paclitaxel given every 3 weeks plus carboplatin (AUC 6 mg/mL per min)

Considering that MITO-7 trial [4] had dissimilar carboplatin administration schedule (AUC 2 mg/mL per min *versus* AUC 6 mg/mL per min), we performed a subgroup analysis for drug dose. Moreover, since the GOG 0262 study design [5] provided bevacizumab to each patient who chose to receive it, only data from the subgroup of patients who elected to not receive bevacizumab were included. Thus, the following sub-group analysis concerned data from GOG 0262 study (without bevacizumab) and JGOG 3016 study, including a total of 743 patients. The benefit of weekly schedule on PFS was statistically significant compared with 3-weekly regimen (HR 0.73, 95% CI 0.61-0.88, *p* = 0.001; I^2^ 0%; Figure 3)

### Overall survival

**Figure 4:**
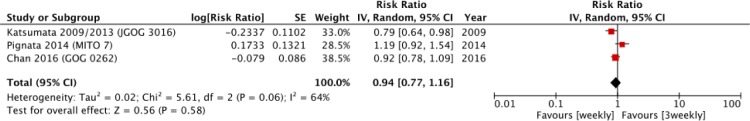
Overall survival of overall cohort

There was no statistically significant beneficial effect for OS comparing weekly *versus* 3-weekly schedule (HR 0.95, 95% CI 0.77-1.16, *p* = 0.06; I^2^ 64%; Figure 4). The effect of both schedules without bevacizumab on OS was not assessable, because GOG-0262 data on OS that included only patients who opted not to receive bevacizumab were not available [5].

### Toxicity

**Figure 5:**
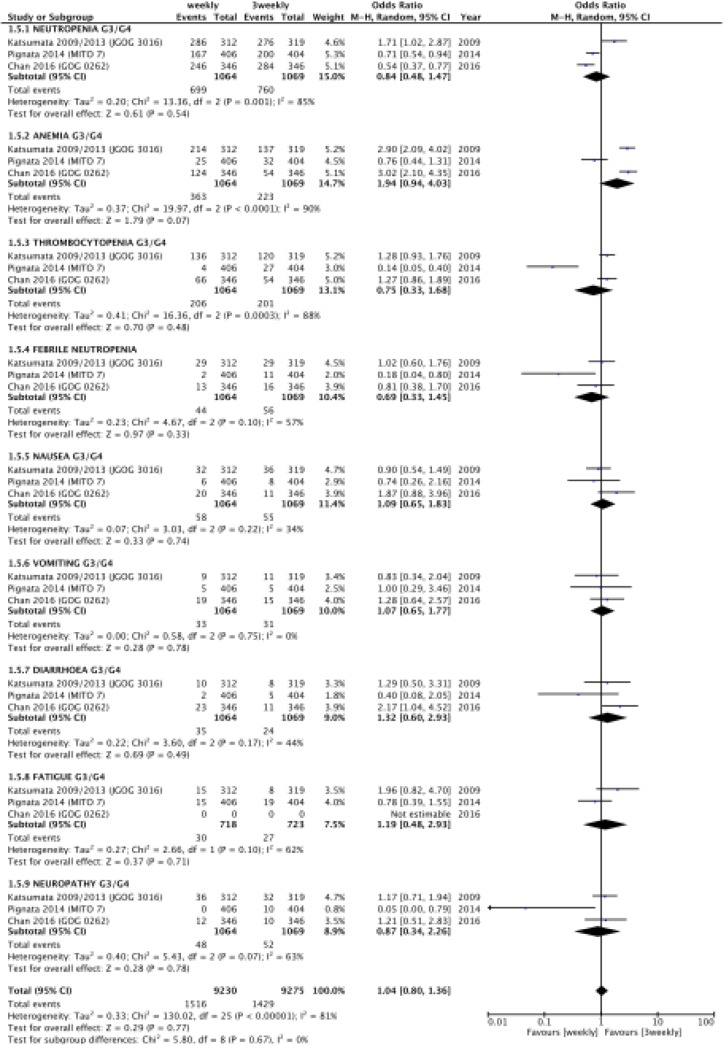
Severe acute toxicity

Overall the incidence of severe acute toxicity was similar between groups. Details are shown in Figure 5. When carboplatin AUC 6 mg/mL per min data were analyzed, the rates of anemia G3-4 (HR 2.95, 95% CI 2.32-3.77, *p* = 0.00001; I^2^ 0%), thrombocytopenia G3-4 (HR 1.28, 95% CI 1.00-1.64, *p* = 0.05; I^2^ 0%) and diarrhea G3-4 (HR 1.78, 95%CI 1.00-3.18, *p* = 0.05; I^2^ 0%) were significantly higher in weekly regimen.

## DISCUSSION

Our meta-analysis demonstrates that, although there was no difference in OS and severe acute toxicity, carboplatin plus weekly paclitaxel administration significantly improves PFS in frontline treatment of advanced OC (HR 0.87, 95% CI 0.76-0.99). In particular, we found a clear advantage when chemotherapy was administered as carboplatin AUC 6 mg/mL per min every 3 weeks and weekly paclitaxel 80 mg/m^2^ (HR 0.73, 95% CI 0.61-0.88). This is considered the “standard” experimental regimen and is named dose-dense as in literature.

This meta-analysis was initially performed to clarify whether dose-dense chemotherapy might enhance the effect of chemotherapy in the first line treatment of OC, with improved toxicity profile, as it had been shown in other cancers [8].

Several randomized trials addressing this question have been conducted in OC but study design and dose administration were partly heterogeneous and, accordingly, results were controversial. Thus, we tried to focus on the dose-dense schedule which has obtained the greatest improvement - carboplatin AUC 6 mg/mL per min every 3 weeks and weekly paclitaxel 80 mg/m^2^ -. This dose-dense schedule was beforehand investigated by Katsumata et al [7]. They found an impressive longer median PFS (28.2 months versus 17.5 months; *p* = 0·0037) as well as median OS (100.5 months versus 62.2 months; 0.039) in those patients who received dose-dense experimental combination compared with conventional chemotherapy. Nonetheless, these data were claimed to be limited to the Japanese population which has been shown to have a significantly better survival compared with non-Hispanic white patients [9–10]. With this regard, duPont et al [9] enrolled patients from South Korea and Japan in the GOG 218 trial and found a significantly longer OS in Asian patients when adjusted for age, stage, residual disease, performance status, and histology. These data need to be confirmed in prospective trial, aimed to explore biological differences, environmental factors, socioeconomic factors, and response to treatment. Nonetheless it should be underlined that when looking at those patients from the GOG 0262 trial who did not received bevacizumab, data about PFS advantage are extraordinary similar in terms of PFS improvement (HR 0.62, 95% CI 0.40 - 0.95) [5], thus justifying the advantage we found in our meta-analysis. Therefore, we can assume that in terms of absolute months of OS and PFS, Asiatic population might have a definitive and ethnic-specific advantage, but the reason for the PFS increment across the two different populations - Asiatic in the JGOG 3016 and non-Hispanic white patients in GOG 0262 - has to be searched not in the ethnicity but in the schedule of treatment received.

We did not found any survival advantage in the OS analysis. Nonetheless, as for toxicity, OS data where not homogenous, since results from the GOG 0262 trial where limited to aggregate analysis, without splitting OS data between those who and who did not received bevacizumab. It might be supposed that the anti-angiogenic properties of bevacizumab might have overlap the anti-angiogenic activity of weekly paclitaxel as well as tumor perfusion and drug delivery, and this might have confused the peculiar efficacy of dose dense administration. Considering that the OS results approached statistical significance (*p* = 0.06), one may speculate that the present meta-analysis might be underpowered to identify a small, but clinically important OS benefit. Therefore, we acknowledge that the present OS results may not be seen as definitive due to the small number of trials presently available. Data from ICON8 and ICON8b trials are currently ongoing and addressing these questions [11].

Regardless, it should also be underlined that in each trial included in our analysis, the PFS was the primary end point of the study. The debate on the role of PFS as primary endpoint in OC clinical trials has been longley discussed and in the last years it has become to be considered as a surrogate endpoint of OS. The advantages of this endpoint are an earlier and more sensitive assessment of antitumor efficacy, a lower likelihood of influence by competing risks (especially in elderly subjects), and a lesser chance of confounding because of treatments received after progression.

Unfortunately we were unable to present toxicity data limited to patients who received dose-dense paclitaxel, without other drugs. In fact, it was not possible to distinguish between patients receiving bevacizumab form those only receiving doublet combination, in the GOG 0262 trial [5]. Interestingly, when looking to cumulative toxicity data, we noticed an increase of anemia, thrombocytopenia and diarrhea in those who received dose-dense schedule. Whether or not these adverse events have to be more related with bevacizumab is not possible to define; nevertheless our toxicity results were consistent with previous findings [8], even if partially in contrast with the initial expectation of lower toxicity rates with a dose-dense schedule. It is reasonable to hypothesize that the greater number of infusions and longer duration of paclitaxel exposure with a dose-dense regimen enhance intratumoral drug perfusion and inhibit angiogenesis, achieving a mechanism of efficacy and a toxicity profile comparable to that of bevacizumab. Furthermore, it appears that quality of life, which has been investigated in these trials [4–5, 12], is not significantly impaired by dose-dense administration, thus suggesting that even if more toxic, this kind of schedule is overall tolerable and manageable.

Our meta-analysis has some limitations. As stated above, none of the eligible trials was designed to measure the OS as primary outcome. Furthermore, our meta-analysis was based on data from trials that have published results in the literature. The use of updated individual patient data might further enhance the accuracy and reduced the uncertainty of the observed estimates [13–14]. However, we made an effort to include all additional information that we could obtain and definitions for the analyzed outcomes were largely consistent across studies. Another limitation of this meta-analysis is that there was heterogeneity in the design, modes of treatment used in each study. Due to the notable differences in the study design and regimens used it is difficult to reach firm conclusions. Finally we should consider that the cohort of patients receving dose-dense schedule is rather small and larger population would be helpful to confirm our results.

Allowing for these cautions, our meta-analysis provides evidence that dose-dense regime results in consistent PFS advantage. We believe that these results provide further insight into the argument concerning the optimal dose and schedule of front line therapy in advanced OC. More randomized studies with better drug use and patient selection are needed; next studies should also clarify which is the role of bevacizuamb in the dose-dense schedule. If our findings will be confirmed, dose-dense schedule might become a valid approach for advanced OC treatment.

Dose-dense regimen, based on carboplatin AUC 6 mg/mL per min every 3 weeks and weekly paclitaxel 80 mg/m^2^ seems to guarantee an important PFS benefit in patients with advanced OC. Its precise superiority in the management of OC remains to be determined.

## MATERIALS AND METHODS

### Data extraction and trials selection

The preferred reporting items for systematic reviews and meta-analyses (PRISMA) guidelines were followed to perform search strategy and selection processes. The meta-analysis included trials without any restrictions on publication date. The last search was carried out on April 2016. Systematic literature electronic search was conducted in Pubmed, Medline and Scopus databases. The search term used were “randomized”, “dose-dense”, “paclitaxel”, “carboplatin”, “3 weeks”, “weekly” and “ovarian cancer” in the title. These key words were variably combined: 1. “randomized paclitaxel carboplatin weekly 3 weeks ovarian cancer”; 2. “randomized dose-dense paclitaxel carboplatin weekly ovarian cancer”. The inclusion criteria included randomized controlled trials that compared carboplatin plus weekly paclitaxel with standard schedule of carboplatin plus paclitaxel every 3 weeks in OC. Randomized clinical trials, written in English, were included, without any restrictions on publication date. We excluded trials meeting the following criteria: non-randomized, single-arm phase II trial or adequate statistical analysis information missing. Reference lists of previously published reviews and meta-analysis were explored. Review articles, case reports, commentaries, and letters were not included, and meeting abstracts were not considered because of the insufficient data provided by the authors.

Two independent reviewers (CM and FDF) selected the identified studies based on the title and abstract. If the topic of the study could not be ascertained from its title or abstract, the full-text version was retrieved for evaluation. Disagreement was resolved by discussion or consensus or with a third party (LM). Trials were eligible if participants were newly diagnosed, with histologically or cytologically proven epithelial ovarian, fallopian tube, or primary peritoneal cancer at study entry. In closer evaluation of potentially eligible articles, when two articles appeared to report results with overlapping data, only the data representing the most recent publication were included in the meta-analysis.

The extracted data for each trial were recorded into standardized database according to the following parameters: first author's surname, year of publication, trial acronym, sample size of weekly and 3 weeks group, chemotherapy regimen, drug and dosage.

### Endpoints

The primary end points were progression free survival (PFS) and overall survival (OS). Severe acute toxicity was secondary end point.

The definition of both PFS and OS was similar across trials. PFS was defined as the time from the date of randomization to last follow-up, death or disease progression. OS was defined as the time from the date of randomization to last follow-up or death. The hazard ratio (HR) and the number of events (death and progression), when available, were derived from each study. At least one of these two outcomes should have been assessed and reported in the trial to be included in the present analysis. Among trials reporting the results of different therapeutic approaches, when possible, we selected and included in a subgroup analysis only the groups of patients who underwent to similar strategies.

We also planned to analyze severe acute toxicity. The following adverse effects grade ≥ 3 were recorded: neutropenia, thrombocytopenia, anemia, febrile neutropenia, vomiting, diarrhea, fatigue and neuropathy.

### Statistical analysis

Statistical analysis was performed using Review Manager 5.0 (http://www.cochrane.org). The pooled HR was calculated using a random-effects model. Forest plots were used for graphical representation of each study and pooled analysis. The size of each box represents the weight that the corresponding study exerts in the meta-analysis; confidence intervals (CIs) for each study are displayed as a horizontal line through the box. The pooled HR is symbolized by a solid diamond at the bottom of the forest plot, and the width of the square represents the 95 % CI of the HR. HR, variance, 95 % CI, log [risk ratio] and standard error for each study were extracted or calculated, based on the published studies, according to the methods described by Tierney et al. in 2007 [15]. A significant two-way p value for comparison was defined as *p* < 0.05. Statistical heterogeneity among studies was examined using both the Cochrane Q statistic (significant at *p* < 0.1) and the I^2^ value (significant heterogeneity if > 50 %) [16]. Publication bias was examined using analyses described by Egger et al.[17] and Begg et al [18].
